# Inhibition of Cdc42 is essential for Mig-6 suppression of cell migration induced by EGF

**DOI:** 10.18632/oncotarget.10205

**Published:** 2016-06-21

**Authors:** Xinni Jiang, MengMeng Niu, Deshi Chen, Jing Chen, Yang Cao, Xiaorong Li, Haoqiang Ying, Johann Bergholz, Yujun Zhang, Zhi-Xiong Xiao

**Affiliations:** ^1^ Center of Growth, Metabolism and Aging, Key Laboratory of Bio-Resource and Eco-Environment of Ministry of Education, College of Life Sciences, Sichuan University, Chengdu, 610014, China; ^2^ Current address: The University of Texas MD Anderson Cancer Center, Houston, TX 77030, USA; ^3^ Current address: Department of Cancer Biology, Dana-Farber Cancer Institute, Boston, MA 02115, USA

**Keywords:** Mig-6, Cdc42, EGF, migration

## Abstract

The adaptor protein Mig-6 is a negative regulator of EGF signaling. It is shown that Mig-6 inhibits cell migration via direct interaction with the ErbB receptors, thereby inhibiting cross-phosphorylation or targeting the receptors for degradation. Mig-6 has also been shown to bind to and inhibit the Rho GTPase Cdc42 to suppress cytoskeletal rearrangement. However, the molecular mechanism(s) by which Mig-6 inhibits cell migration via Cdc42 is still not entirely clear. Here, we show that Mig-6 binding to Cdc42 is necessary and sufficient to inhibit EGF-induced filopodia formation and migration. This binding, mediated by four specific residues (I11, R12, M26, R30) in the Mig-6 CRIB domain, is essential for Mig-6 function. In addition, ectopic expression of Cdc42 reverses Mig-6 inhibition of cell migration. Mig-6 CRIB domain, alone, is sufficient to inhibit cell migration. Conversely, Mig-6 binding to EGFR is dispensable for Mig-6-mediated inhibition of cell migration. Moreover, we found that decreased Mig-6 expression correlates with cancer progression in breast and prostate cancers. Together, our results demonstrate that Mig-6 inhibition of Cdc42 signaling is critical in Mig-6 function to suppress cell migration and that dysregulation of this pathway may play a critical role in cancer development.

## INTRODUCTION

Mitogen-inducible gene-6 (Mig-6; also known as RALT, Errfi1 and Gene-33) encodes a cytosolic adaptor protein that is evolutionarily conserved in vertebrates [1] and is implicated in suppression of tumorigenesis, cancer progression, and metastasis [2]. The *Mig-6* gene locus on chromosome 1p36 is frequently deleted in lung cancers [3–5]. Mig-6-null mice exhibit spontaneous tumor formation in multiple tissues, including the lungs, gallbladder, and bile duct [6–8].

The Mig-6 protein consists of several protein-protein interaction domains, including an N-terminal Cdc42/Rac-interaction and binding (CRIB) domain, Src-homology 3 (SH3)-binding moieties, a 14-3-3 protein-binding motif, and an Ack1 homology (AH) domain, which contains an Epidermal Growth Factor Receptor (EGFR)-binding segment [8, 9]. Mig-6 has been shown to interact with and inhibit all four ErbB family members. Previous studies showed that Mig-6 binds to the catalytic domain of EGFR (also known as ErbB1), ErbB2 (also known as Her2), ErbB4, or ErbB2-ErbB3 heterodimers [10–12] to inhibit receptor autophosphorylation and catalytic activity upon ligand binding [9, 13, 14]. More recently, Mig-6 was shown to induce EGFR endocytosis and lysosomal degradation in glioblastoma cells via direct interaction with the SNARE protein STX8 [15]. Moreover, Mig-6 expression can be stimulated by EGF, thus acting as a negative feedback regulator to restrain EGF signal strength and duration [10, 11, 16, 17].

EGF signaling plays a pivotal role in tumorigenesis, cancer progression, and metastasis. ErbB family members activate both the Ras-MAP Kinase and the PI3K-Akt cascades, thus promoting cell survival, proliferation, migration, and invasion [18]. Gain- and loss-of-function studies have clearly shown that Mig-6 inhibits ErbB family signaling [6, 10–12, 17]. Indeed, ectopic expression of Mig-6 in mammary epithelial cells leads to increased sensitivity to treatment with Herceptin, a recombinant antibody that binds to the ErbB2 extracellular domain and inhibits ErbB2 signaling [19].

At present, Mig-6 tumor suppressor functions have been shown to be mediated via inhibition of EGF signaling at the receptor level. However, the Mig-6 CRIB domain also interacts with Cdc42 protein (a homolog of the yeast cell division control protein 42), a member of the Rho family involved in actin remodeling, chemotaxis and cell migration, and filopodia formation [20]. In this study, we show that Mig-6 inhibits EGF-induced cell migration and Cdc42-mediated actin remodeling, independent of EGFR binding. Thus, our study reveals a novel molecular mechanism by which Mig-6 modulates cell migration.

## RESULTS

### Mig-6 inhibits EGF-induced cell migration and filopodia formation

**Figure 1:**
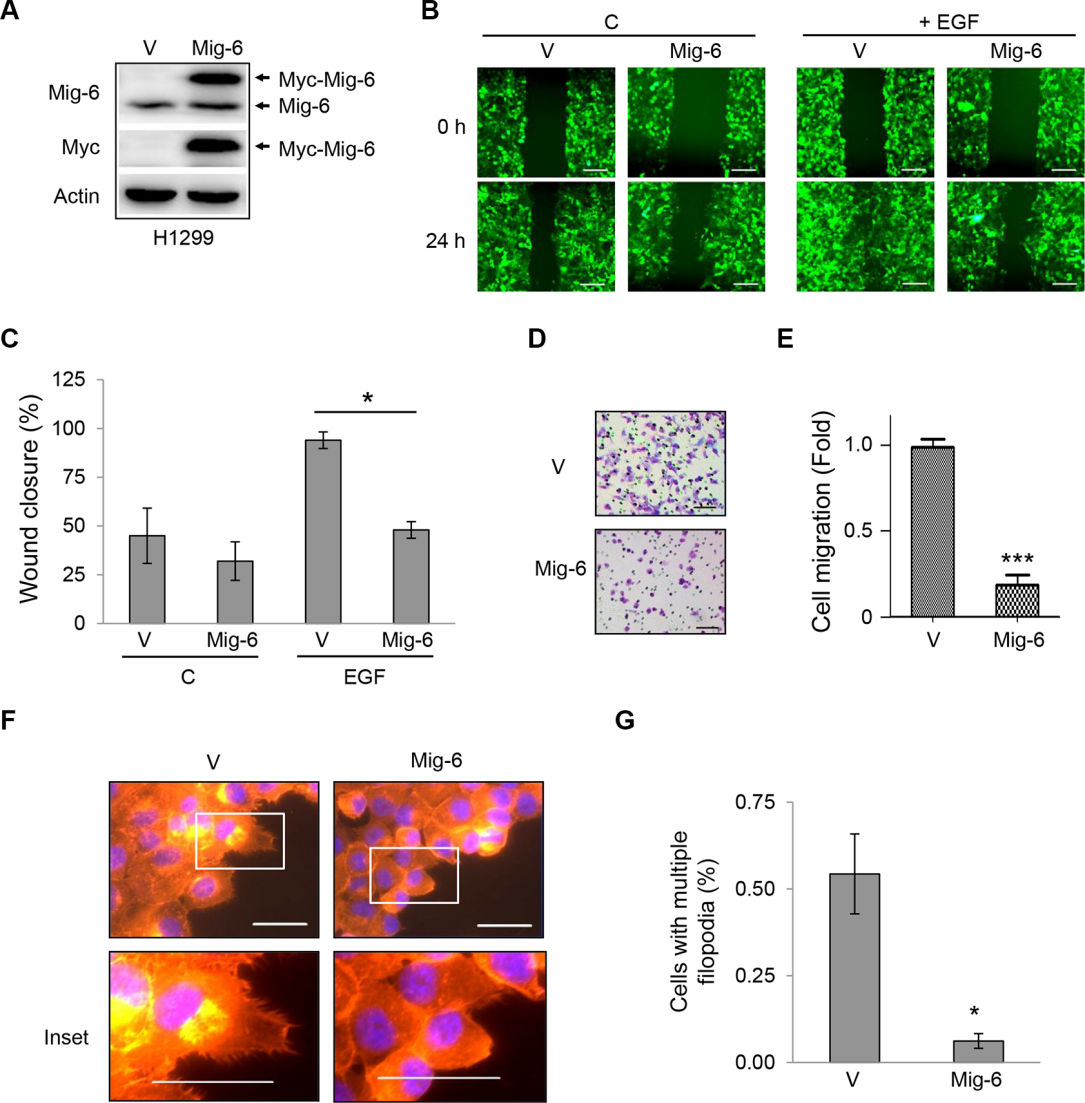
Mig-6 inhibits EGF-mediated cell migration and filopodia formation H1299 cells were stably infected with lentivirus expressing GFP and Myc-tagged Mig-6 (Mig-6), or GFP alone (VEC). (**A**) Whole-cell lysates from these stable cells were subjected to immunoblotting, as indicated. (**B**) Migration of these stable cells was assessed by wound-healing assay in the presence or absence of EGF (100 ng/mL). Scale bars = 200 μm. (**C**) Wound closure data was quantitated and presented as means and SE from three independent experiments. *P < 0.05. (**D**) Stable H1299 cells were subjected to cell migration assays using transwell systems in the presence of EGF. Migratory cells were fixed, stained with crystal violet, and photographed under a light microscope. Representative pictures are shown. Scale bars = 100 μm. (**E**) Results from migration assays were quantitated and presented as means and SE from three independent experiments. ***P < 0.001. (**F**) Stable H1299 cells were grown in the presence of EGF for 24 hours. Cells exhibiting filopodia were visualized by fluorescent microscopy. F-actin was visualized with phalloidin (red), and nuclei was counter-stained with DAPI (blue). Note that Mig6 inhibited EGF-induced filopodia formation. Representative fluorescent microscopic images and insets are shown. Scale bars = 50 μm. (**G**) Percentage of cells with multiple filopodia (defined as cells exhibiting at least 20 filopodia) were quantified and presented as means and SE from three independent experiments performed in duplicate. *P < 0.05.

In order to elucidate the molecular mechanisms by which Mig-6 regulates cell migration, we sub-cloned Myc-tagged Mig-6 (Myc-Mig-6) into a pLVX-IRES-zGreen1 plasmid, which allows bicistronic expression of GFP and Mig-6 so that transfected cells can be visualized with the use of a fluorescent microscope. We established human non-small cell lung carcinoma H1299 cell line stably expressing Myc-Mig-6 or a vector control. Endogenous Mig-6 and exogenous Myc-Mig-6 proteins were confirmed by western blotting (Figure 1A). These stable cells were then subjected to wound-healing assays to examine the effect of Mig-6 expression on cell migration. Myc-Mig-6-expressing and control cells exhibited small but clear reduced migration 24 hours after wounding in the absence of EGF stimulation (Figure 1B, top panel, and 1C). However, EGF treatment significantly stimulated cell migration in the control cells, leading to a near complete closure of the wound after 24 hours, while Myc-Mig-6-expressing cells displayed much reduced cell migration upon EGF stimulation (Figure 1B, bottom panel, and 1C). We then confirmed these results by using transwell migration chambers and measuring cell migration in the presence of EGF after eight hours. As shown in Figure 1D and 1E, Myc-Mig-6 expression dramatically inhibited EGF-stimulated cell migration, indicating that Mig-6 is a potent inhibitor of EGF-induced cell migration.

Cell migration requires a highly dynamic reorganization of the actin cytoskeleton, including the formation of cell protrusions at the leading edge, such as lamellipodia and filopodia, and contraction of the lagging edge. Lamellipodia are flat, sheet-like protrusions formed by a network of branched actin filaments, while filopodia are rod-like extensions consisting of tightly bundled actin fibers that originate from the base of lamellipodia and penetrate into the surrounding environment, and may act as guiding cues to direct cell migration [21–23]. Thus, we examined the effects of Mig-6 on the formation of filopodia in cancer cells. Stable H1299 cells were plated on collagen-coated coverslips and stained with rhodamine-conjugated phalloidin to detect filamentous actin (F-actin) and associated motility structures. Upon stimulation with EGF, the majority of control cells exhibited multiple filopodia around the periphery of the cells. In sharp contrast, Mig-6 expression led to a dramatic and significant decrease in filopodia formation (Figure 1F–1G).

### Mig-6 is a physiologic suppressor of EGF-induced cell migration

**Figure 2:**
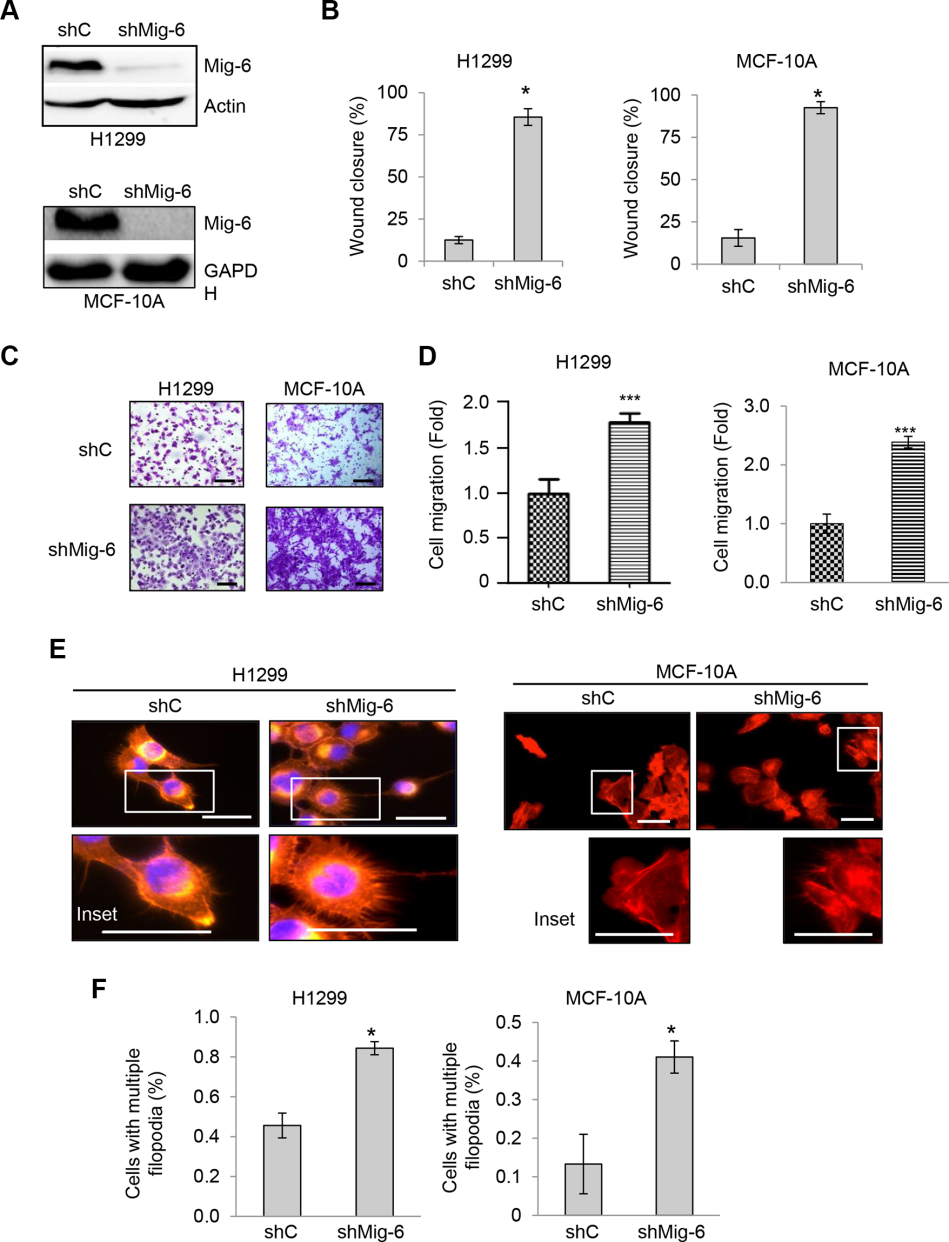
Ablation of Mig-6 enhances EGF-induced cell migration H1299 and MCF-10A cells were infected with lentivirus encoding shRNA specific for Mig-6 (shMig-6) or a scrambled shRNA sequence (shC). (**A**) Cell lysates were subjected to western blotting, as indicated. (**B**) Wound-healing assays were performed in the presence of EGF for 12 hours. Wound closure data was quantitated by percentage of wound closure and presented as means and SE. *P < 0.05. (**C**) Stable H1299 or MCF10A cells were subjected to transwell assays in the presence of EGF for 8 hours (H1299) or 24 hours (MCF10A), respectively. Representative images are shown. Scale bars = 100 μm. (**D**) Results from three independent experiments in duplicate were quantitated and presented as means and SE ***P < 0.001. (**E**) Stable cells exhibiting filopodia were visualized by fluorescent microscopy. F-actin was visualized with phalloidin (red), and nuclei was counter-stained with DAPI (blue). Representative images (and insets) from three independent experiments performed in duplicate are shown. Scale bars = 50 μm. (**F**) Percentage of cells with multiple filopodia (defined as cells exhibiting at least 20 filopodia) were quantified and presented as means and SE from three independent experiments. *P < 0.05.

Since our data showed that exogenous Mig-6 inhibits cell migration, we next examined whether endogenous Mig-6 executes a similar biological function. To this end, we infected H1299 cells and human mammary epithelial MCF-10A cells with lentivirus expressing short hairpin RNA (shRNA) specific for Mig-6 or a scrambled shRNA control (shC) and selected by puromycin resistance. Mig-6 ablation was confirmed by western blotting (Figure 2A). As shown in Figure 2B–2C, silencing of endogenous Mig-6 significantly enhanced EGF-induced cell migration in wound healing assays in both cell types. Similarly, transwell assays also indicated that Mig-6 ablation enhanced migration induced by EGF (Figure 2D–2E). Moreover, reduced Mig-6 expression significantly increased the fraction of cells exhibiting multiple filopodia (Figure 2F). Together, these results indicate that Mig-6 inhibits actin remodeling and results in decreased cell migration upon EGF stimulation.

Next, to investigate the clinical relevance of Mig-6 down-regulation, we analyzed the online microarray database Oncomine for Mig-6 expression in human cancers. From a total of 12 breast cancer transcriptome studies available through Oncomine, we found decreased Mig-6 expression in invasive breast carcinoma in six studies, as well as in three additional cases of lobular or ductal breast carcinoma, compared to normal breast (Supplementary Figure 1A). In addition, we found decreased Mig-6 expression in metastatic lesions compared to samples from primary tumors in prostate cancer (Supplementary Figure 1B). Furthermore, data from The Cancer Genome Atlas (TCGA) showed that Mig-6 expression was decreased in breast cancer samples comparing to normal samples (Supplementary Figure 1C). Notably, there is significant difference of Mig-6 expression between normal samples and samples at different tumor stages, while no significant difference between different tumor stages. These results suggest that reduced expression of Mig-6 may be important for breast tumor initiation.

### Cdc42-binding via CRIB domain is essential for Mig-6 inhibitory function on EGF-induced migration

**Figure 3:**
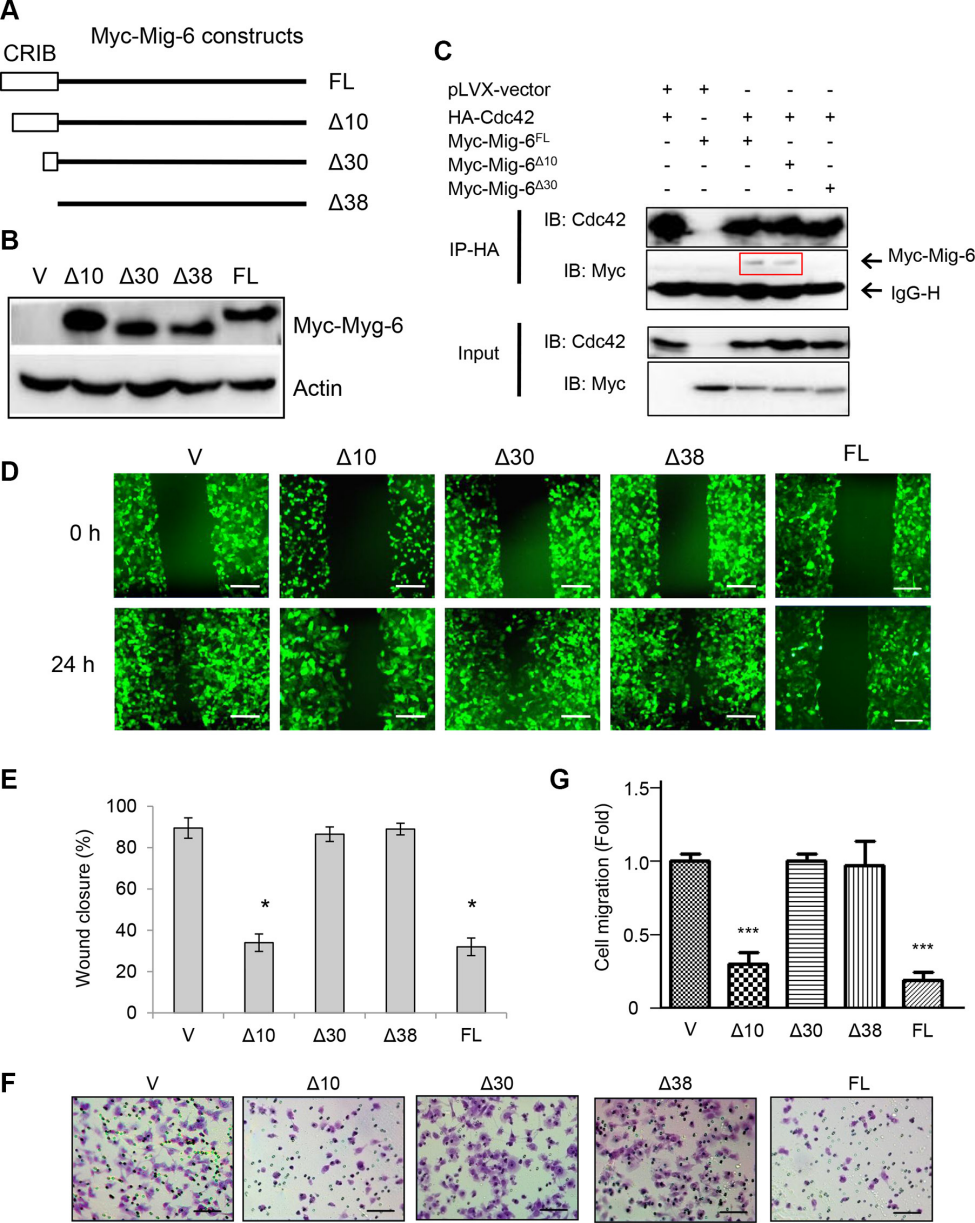
CRIB domain and Cdc42-interaction with Mig-6 is indispensable for Mig-6 inhibitory function on EGF-induced migration (**A**) Schematic representation of Mig-6 deletion mutants (not to scale), including full length Mig-6 (FL), Mig-6 lacking the first ten amino acid residues on the N-terminal CRIB domain (Δ10), Mig-6 lacking the first thirty amino acid residues of the CRIB domain (Δ30), and Mig-6 lacking the complete CRIB domain (Δ38). All Mig-6 constructs containing an N-terminal Myc tag were subcloned in to PLVX-GFP lentiviral vector. (**B**) H1299 cells were stably infected with lentivirus expressing Myc-tagged Mig-6, the deletion mutants, or GFP alone. Cell lysates were subjected to western blotting using antibody for C-Myc, as indicated. (**C**) cell lysates from 293T cells co-transfected with HA-Cdc42 and Myc-Mig-6 (FL) or a deletion mutant were subjected to IP-western analysis, as shown. Note that Δ30 was unable to interact with Cdc42. (**D**) Stable H1299 cells were assessed by wound-healing assay in the presence of EGF. Scale bars = 200 μm. (**E**) Wound-healing assay was quantitated by percentage of wound closure and presented as means and SE. *P < 0.05. (**F**) Stable H1299 cells were subjected to transwell assays in the presence EGF. Scale bars = 100 μm. (G) Results from three independent transwell assays in duplicate were quantitated and presented as means and SE. ***P < 0.001 (Δ30/Δ38 vs. FL).

The CRIB domain of Mig-6 consists of 38 amino acid residues and has been shown to be important for inhibiting cell migration [24]. Thus, to identify the structural determinants of Mig-6 required to suppress cell migration, we constructed Mig-6 deletion mutants lacking either the first ten or thirty amino acid residues (Δ10 and Δ30, respectively), or the entire CRIB domain (Δ38; Figure 3A). Of note, these Mig-6 constructs contained an N-terminal Myc tag to facilitate detection by immunoblotting. Notably, the Rho GTPase family member protein Cdc42 has been shown to be a major regulator of filopodia extensions and maintenance of cell polarity [25–27]. Since previous reports have shown that the CRIB domain alone can bind to Cdc42 [24, 28], we asked whether this interaction is required for Mig-6 function in regulating cell migration. To this end, we first co-expressed HA-tagged Cdc42 with either full-length Myc-Mig-6, or the Δ10 or Δ30 deletion mutants in H1299 cells. We then constructed H1299 cells stably expressing either full-length (FL) or a deletion mutant Mig-6 and confirmed comparable expression by western blotting (Figure 3B). Immunoprecipitation of HA-Cdc42 effectively pulled down both full-length Mig-6 and Mig-6-Δ10, but not Mig-6-Δ30 (Figure 3C), indicating that Cdc42 physically interacts with Mig-6 through this specific binding module (residues 11–30). Also, Mig-6-Δ38 failed to interact with Cdc42 (Supplementary Figure 2). Consistent with our previous results, full-length Mig-6 protein expression inhibited EGF-induced cell migration in wound healing assays (Figure 3D–3E). Deletion of the first ten amino acid residues (Δ10) on the CRIB domain had no effect on the ability of Mig-6 to inhibit EGF-induced cell migration, while deletion of the first thirty amino acid residues (Δ30) led to a complete loss of Mig-6 inhibitory function, similar to the lack of the entire CRIB domain (Δ38; Figure 3D–3E). Data from transwell assays showed similar results (Figure 3F–3G). Together, these results indicate that the Cdc42-binding module (residues 11–30) on the CRIB domain is essential for inhibiting cell migration, and that loss of this interaction leads to a complete abrogation of Mig-6 inhibitory function.

### Ile11, Arg12, Met26 and Arg30 in the CRIB domain of Mig-6 are essential for Mig-6 interaction with Cdc42 and inhibition of cell migration

**Figure 4:**
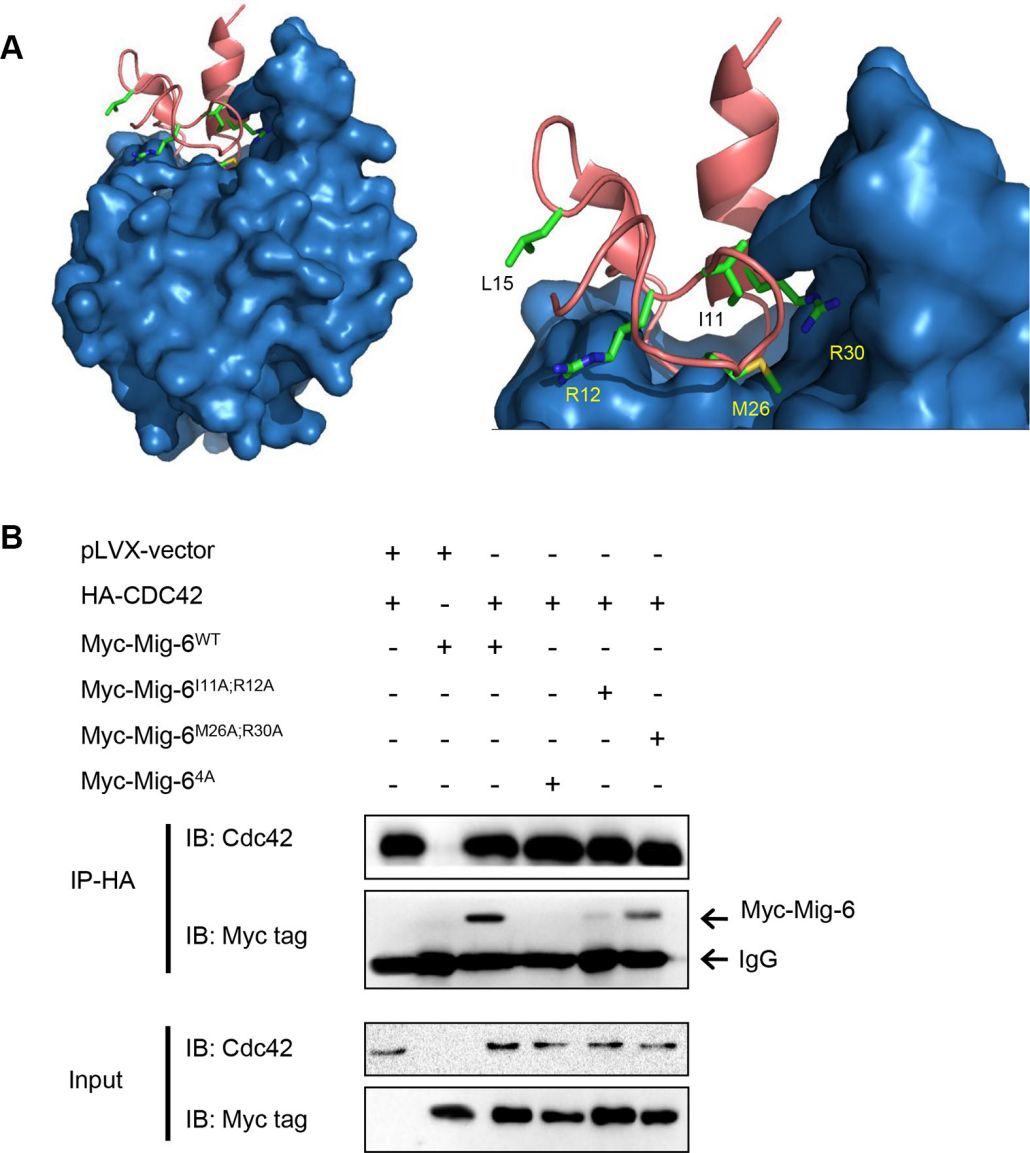
Four amino acid residues (Ile11, Arg12, Met26 and Arg30) in the CRIB domain of Mig-6 are essential for Mig-6 binding to Cdc42 (**A**) Computational modeling of binding interphase between the Mig-6 CRIB domain (pink; top) and Cdc42 (blue; bottom). Amino acid residues Ile11, Arg12, Met26 and Arg30 on Mig-6, which are predicted to mediate interaction with Cdc42, are highlighted. Leu15, which is predicted to not interact with Cdc42, is also highlighted and was used as a control in experiments. (**B**) 293T cells were transiently transfected with HA-tagged Cdc42 and Myc-tagged WT, double point mutant (I11A/R12A or M26A/R30A) or quadruple point mutant (4A [I11A/R12A/M26A/R30A]). Cell lysates were subjected to IP-western analyses, as shown.

Next, we sought to determine which specific amino acid residues in Mig-6 are critical for interacting with Cdc42. We used Protein Docking Software Zdock followed by KFC2 Hot Spot Prediction Server to model the binding interphase between Cdc42 and the first thirty amino acid residues on the CRIB domain of Mig-6. As shown in Figure 4A, computational analysis of the composite structure revealed a putative CRIB binding pocket in Cdc42, as well as four amino acid residues in Mig-6 important for binding: Ile11, Arg12, Met26, and Arg30. We predicted that these four amino acid residues fit in the Cdc42 binding pocket and are important for interacting with Cdc42. Using site-directed mutagenesis, we generated single point mutant Mig-6 derivatives bearing alanine substitutions (I11A, R12A, M26A, and R30A, respectively) or a mutant derivative with all four alanine residues substituted in full length Mig-6(FL-4A). In addition, Leu15, which is found outside the binding pocket, was also mutated to an alanine residue (L15A) as a control. We then examined the ability of the mutant derivatives for binding with Cdc42 by co-expressing HA-Cdc42 and wild type or point-mutant Myc-Mig-6 in H1299 cells, followed by IP-Western analyses. As shown in Figure 4B, wild type Mig-6(WT) exhibited robust interaction with Cdc42, while Mig-6(4A) completely lost the ability to interact with Cdc42. Importantly, mutation of Ile11 and Arg12 on Mig-6 greatly disrupted the Mig-6-Cdc42 interaction, while Mig-6(M26A;R30A) still exhibited a significant ability to interact with Cdc42, albeit markedly weaker than WT Mig-6.

**Figure 5:**
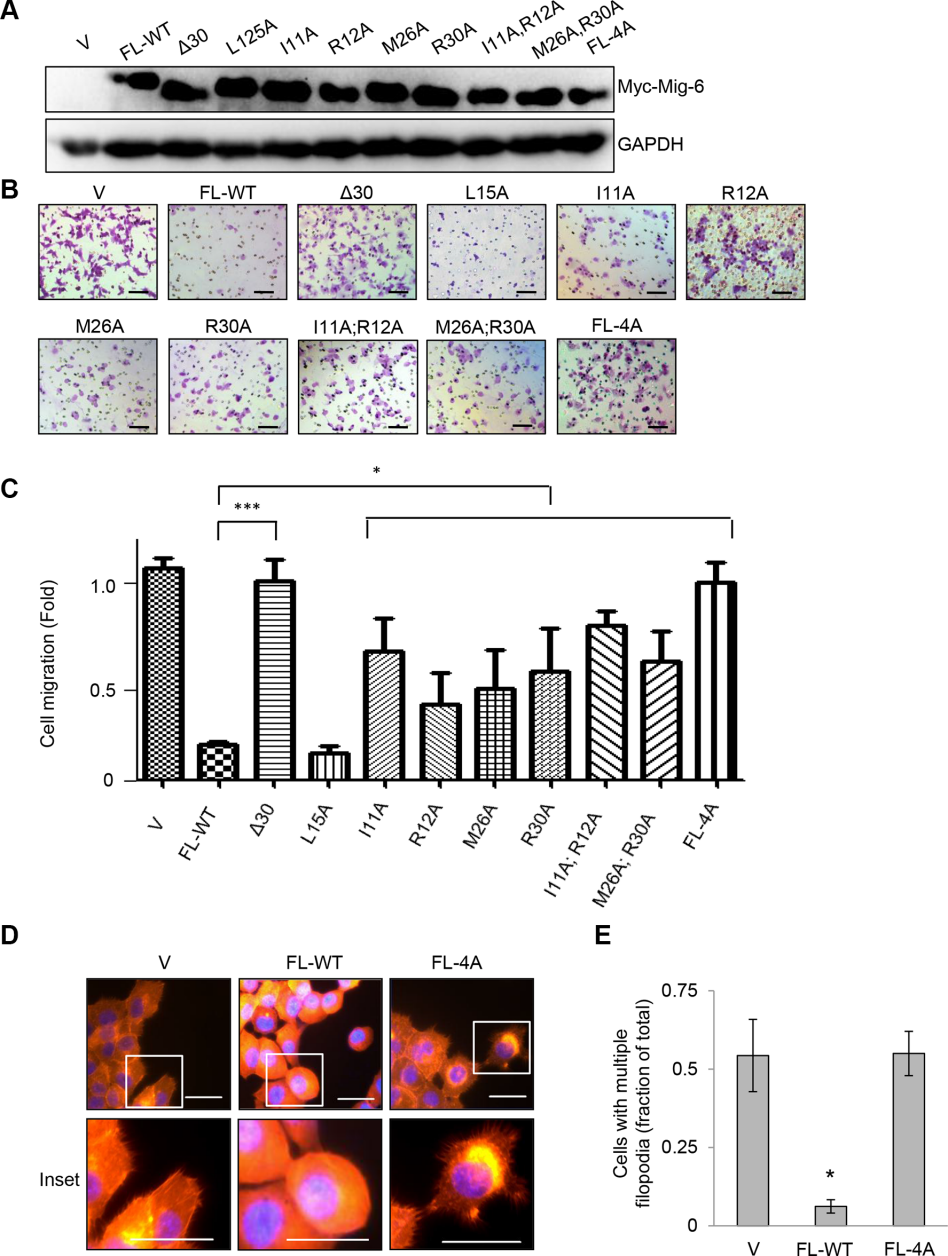
Ile11, Arg12, Met26 and Arg30 in the CRIB domain of Mig-6 are essential for Mig-6 inhibition of EGF-induced cell migration (**A**) H1299 cells stably expressing either full-length Myc-Mig-6 (FL), a deletion mutant Myc-Mig-6 (Δ30) or point mutant Myc-Mig-6 derivatives were subjected to western blotting as indicated. (**B**) Stable H1299 cells expressing either wild type or mutant Myc-Mig-6 were subjected to transwell assays in the presence of EGF for 8 hours. Representative images are shown. Scale bars = 100 μm. (**C**) Results from three independent migration assays were quantitated and presented as means and SE. *P < 0.05. (**D**) Stable cells exhibiting filopodia were visualized by fluorescent microscopy. F-actin was visualized with phalloidin (red), and nuclei was counter-stained with DAPI (blue). Representative images (and insets) from three independent experiments performed in duplicate are shown. Scale bars = 50 μm. (**E**) Percentage of cells with multiple filopodia (defined as cells exhibiting at least 20 filopodia) were quantified and presented as means and SE from three independent experiments. *P < 0.05.

Next, we analyzed the effects of these point mutations on Mig-6-mediated inhibition of cell migration upon EGF stimulation. Each mutant derivative was expressed comparable to wild type full-length Myc-tagged Mig-6 (FL-WT), as assayed by western blotting (Figure 5A). As shown in Figure 5B–5C, while FL-WT Mig-6 abrogated EGF-induced cell migration, the Δ30 deletion mutant was unable to do so. On the other hand, Mig-6(L15A) did not affect the ability of Mig-6 to inhibit cell migration, whereas all other single point mutation (I11A, R12A, M26A, or R30A) resulted in a significant, albeit incomplete loss of inhibition for cell migration upon EGF stimulation. Double point mutations (I11A;R12A or M26A;R30A) further impinged on the ability of Mig-6 to inhibit EGF-stimulated cell migration, whereas mutation of all four sites to alanine (FL-4A) completely abrogated the ability of Mig-6 to inhibit cell migration. Moreover, while FL-WT Mig-6 inhibited filopodial extensions in H1299 cells, cells expressing FL-4A displayed numerous filopodia throughout the periphery of the cells, similar to control cells (Figure 5D–5E). These results indicate that the four amino acids residues (Ile11, Arg12, Met26 and Arg30) are essential for Mig-6 interaction with Cdc42 and for Mig-6 function in suppression cell migration.

### Mig-6 CRIB domain binding to Cdc42 is necessary and sufficient to inhibit cell migration independent of EGFR binding

**Figure 6:**
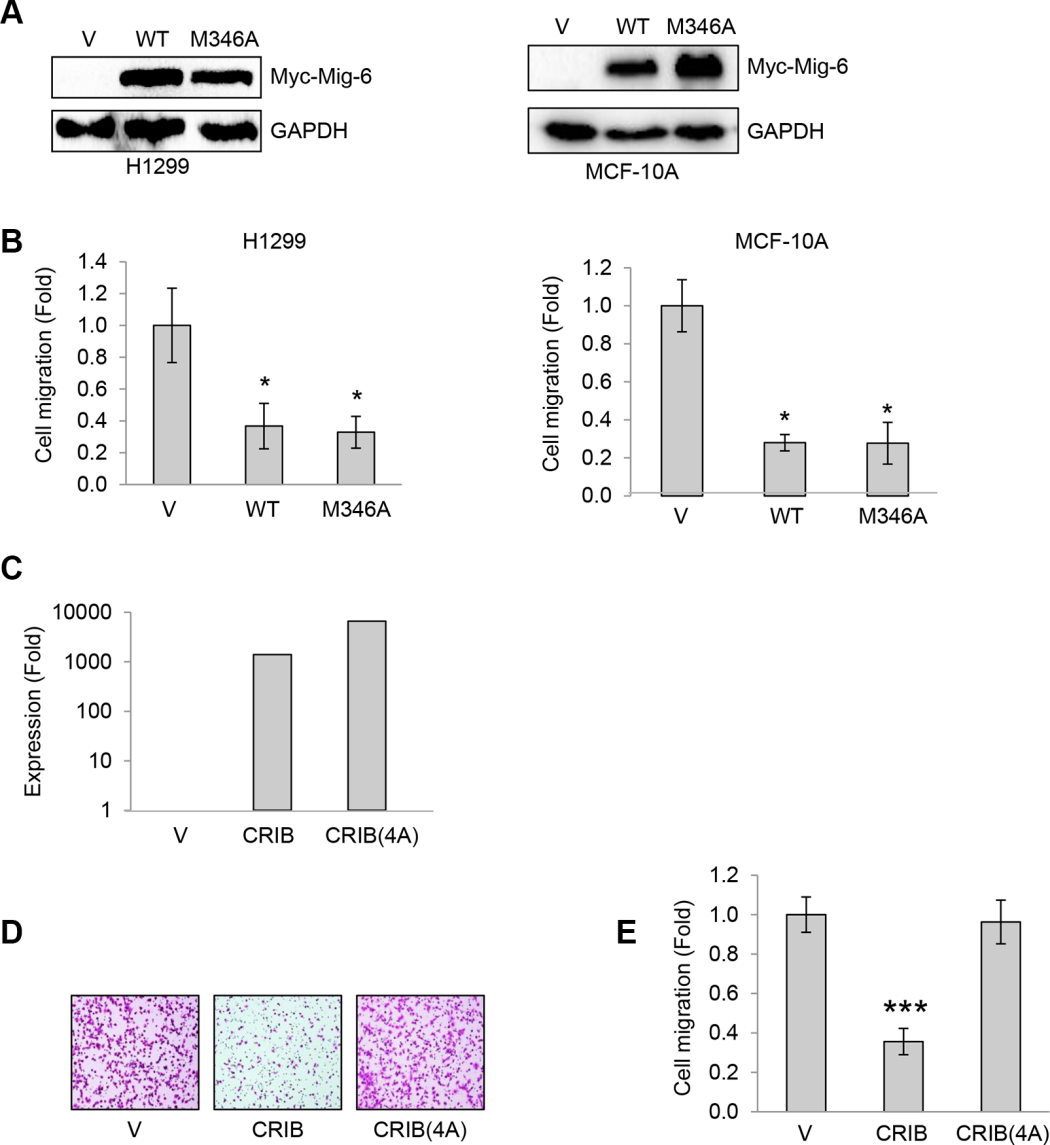
Mig-6 CRIB domain binding to Cdc42 is necessary and sufficient to inhibit cell migration independently of EGFR binding (**A**) H1299 cells and MCF-10A cells were stably infected with lentivirus expressing wild type (WT) or EGFR-binding deficient mutant Myc-tagged Mig-6(M346A). Whole cell lysates were subjected to western blotting as shown. (**B**) Stable H1299 and MCF-10A cells were subjected to transwell assays in the presence of EGF for 8 hours (H1299) or 24 hours (MCF10A), respectively. Results from three independent migration assays were quantitated and presented as means and SD. *P < 0.05. (**C**–**D**) H1299 cells stably expressing with CRIB or a quadruple mutant (CRIB-4A) were subjected to q-PCR analyses (C), or transwell assays in the presence of EGF for 8 hours. Representative images are shown (D). (**F**) Results from three transwell assays in duplicate were quantitated and presented as means and SD. ***P < 0. 001.

Since Mig-6 has been shown to inhibit EGF signaling by binding to the EGF receptor (EGFR), we examined the effects of an EGFR binding-deficient mutant Mig-6 (M346A), which does not bind to the kinase domain of EGFR (Zhang et al., 2007a), for its ability of inhibiting EGF-stimulated cell migration. As shown in Figure 6, both WT Mig-6 and Mig-6(M346A) inhibited migration of H1299 cells and MCF-10A cells equally well (Figure 6A–6C), suggesting that Mig-6-mediated inhibition of cell migration does not require Mig-6 binding to EGFR. In addition, two other Mig-6 EGFR-binding mutants(F352A, Y358A) also inhibited cell migration, similar to that of WT Mig-6 (Supplementary Figure S3).

We next examined the effects of Mig-6(4A) mutants on EGFR phosphorylation. As shown in Supplementary. Figure S4, expression of Mig-6(4A) led to a clear reduction of both EGFR protein levels and EGFR phosphorylation. Moreover, expression of wild type Mig-6 CRIB domain alone (CRIB), but not CRIB(4A), was sufficient to inhibit H1299 cell migration (Figure 6D–6E). Together, these data indicate that Mig-6 CRIB domain binding to Cdc42 is necessary and sufficient to inhibit cell migration independently of EGFR binding.

In addition, we examined whether WT and mutant Mig-6 affected cell proliferation. Expression of WT Mig-6 led to slower cell growth, consistent with Mig-6 role in inhibiting mitogenic signaling. Notably, Cdc42 binding-deficient Mig-6(4A) and EGFR binding-deficient Mig-6(M346A) inhibited cell proliferation to similar extents, albeit to a lesser extent than WT Mig-6 (Supplementary Figure S5). These results suggest that, in addition to inhibiting cell migration, Mig-6 binding to Cdc42 may be also involved in cell proliferation.

### Mig-6 inhibits Cdc42 activation and downstream PAK-1

**Figure 7:**
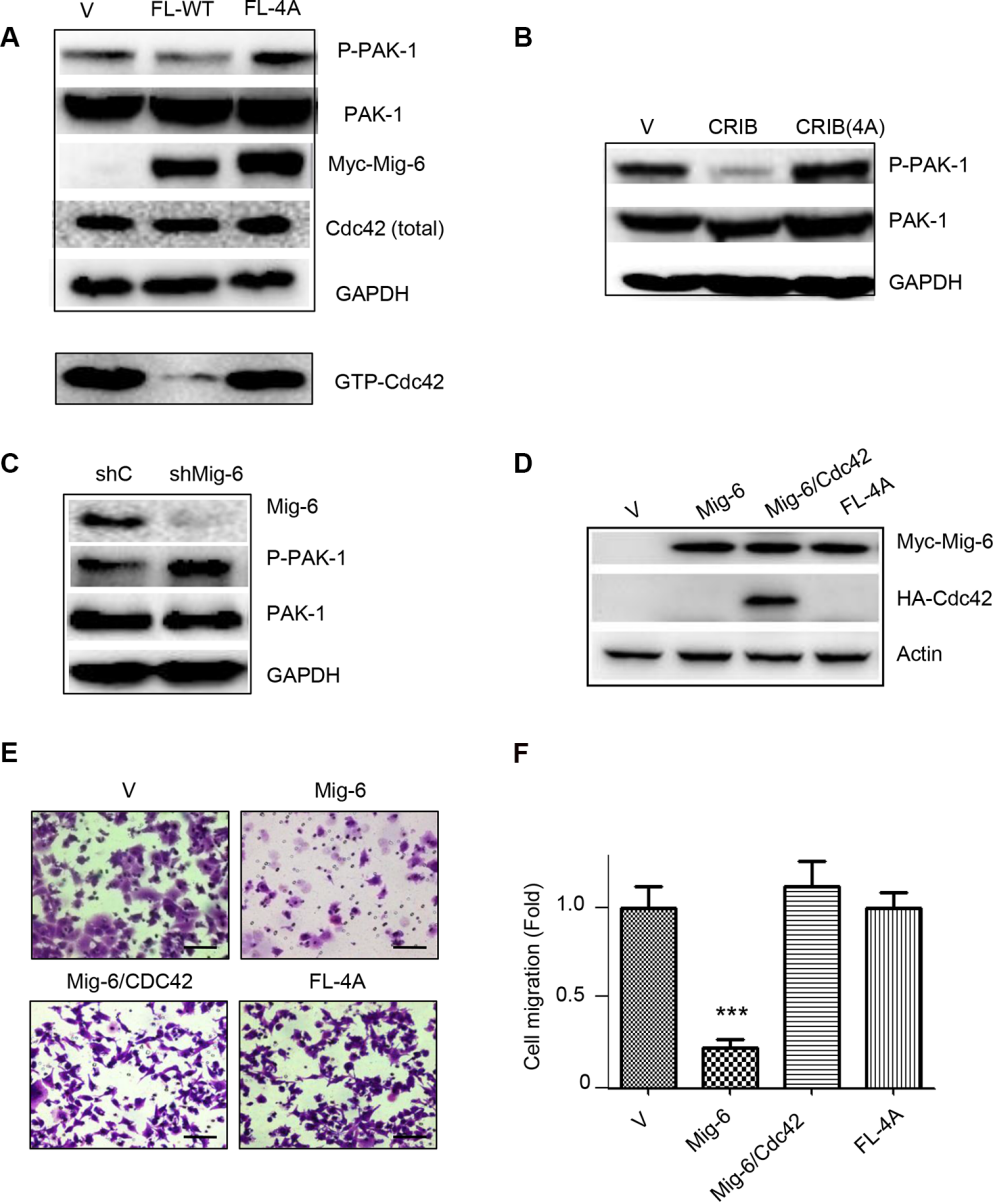
WT Mig-6, but not the 4A mutant, inhibits Cdc42 activation (**A**) H1299 cells stably expressing Mig-6-FL or FL-4A mutant were subjected to western blotting, as shown. (**B**) H1299 cells stably expressing CRIB or CRIB-4A were subjected to western blotting. (**C**) H1299 cells stably expressing shRNA specific for Mig-6 (shMig-6) or a scrambled shRNA sequence (shC) were subjected to western blotting. (**D**) Ectopic expression of Cdc42 reverses Mig-6 inhibitory effect on EGF-induced cell migration. H1299 cells stably expressing Myc-Mig-6 were infected with lentivirus expressing HA-Cdc42 or vector control. H1299 cells stably expressing mutant Myc-Mig-6(4A) was used in parallel. Cell lysates were subjected to western blotting, as indicated. (**E**) Stable cells were subjected to transwell assays in the presence of EGF for 8 hours. Representative pictures are shown. Scale bars = 100 μm. (**F**) Results from three independent migration assays were quantitated and presented as means and SE. ***P < 0.001.

We next examined whether Mig-6 binding to Cdc42 affected Cdc42 activation and downstream signaling. Expression of WT Mig-6, but not Mig-6(4A), led to decreased levels of GTP-bound Cdc42 (Figure 7A). Furthermore, expression of Mig-6 or Mig-6-CRIB domain, alone, markedly inhibited PAK-1 activation, as evidenced by decreased phosphorylation of PAK-1(Ser144). By contrast, Mig-6(4A) and CRIB(4A) expression did not affect PAK-1 phosphorylation (Figure 7A–7B). Moreover, silencing of endogenous Mig-6 by shRNA resulted in increased PAK-1 phosphorylation in H1299 cells (Figure 7C). since PAK-1 is a major downstream effector of Cdc42 [29, 30], these results suggest that Mig-6 binds to Cdc42 and inhibits Cdc42 downstream signaling.

To demonstrate that Mig-6-Cdc42 axis is responsible for Mig-6 suppression of EGF-induced cell migration, we investigated whether expression of Cdc42 can rescue Mig-6-mediated suppression of cell migration induced by EGF. As shown in Figure 7F, again, WT Mig-6 significantly inhibited cell migration, while Mig-6(4A) was unable to do so. Mig-6-mediated inhibition of cell migration and filopodia formation was completely reversed by ectopic expression of Cdc42 (Figure 7E–7F; and Supplementary Figure 6). Together, these results indicate that Mig-6-mediated inhibition of cell migration upon EGF stimulation is dependent on Mig-6-Cdc42 interaction and inhibition of Cdc42 signaling.

## DISCUSSION

Mig-6 has been described as a tumor suppressor protein important for inhibiting metastasis in a variety of human cancers [2, 8]. In this study, we demonstrate that Mig-6 interaction with Cdc42 confers Mig-6 suppression function in EGF-induced cell migration. First, a direct physical interaction between Mig-6 and Cdc42 is essential for Mig-6 function towards inhibiting EGF-induced filopodia formation and cell migration. Second, the Mig-6 CRIB domain alone is necessary and sufficient to inhibit Cdc42-PAK-1 signaling and cell migration. Third, four specific amino acid residues (Ile11, Arg12, Met26 and Arg30) on the CRIB domain of Mig-6 are critical for binding to Cdc42 protein and for Mig-6 function. Finally, ectoic expression of Cdc42 can effectively rescue Mig-6 suppressive function. This study indicates that, in addition to direct inhibition of EGFR, Mig-6 can function to inhibit Cdc42, and that dysregulation of Cdc42 pathway may play an important role in cancer metastasis.

EGF signaling is up-regulated in human cancers, including approximately 25% of breast cancer cases, as well as subsets of ovarian, lung, gastric and salivary gland tumors [31–38]. EGF receptor internalization and degradation leads to irreversible inhibition of EGF signaling [39]. Importantly, Mig-6 has been shown to act directly at the receptor level both reversibly by blocking catalytic activity [10–12], and non-reversibly by targeting the receptor to lysosomal degradation. By doing so, Mig-6 inhibits cell growth and proliferation, as well as cell invasion [15].

In this study, we show that, like WT Mig-6, EGFR binding mutants of Mig-6 (M346A, F352A and Y358A) can inhibit migration. On the other hand, like WT Mig-6 [15], Cdc42-binding mutant of Mig-6, Mig-6(4A), down regulates EGFR protein level and as well as EGFR phosphorylation. Together, these results indicate that Mig-6 function to inhibit EGF-induced cell migration is independent of EGFR-binding. Rather, the CRIB domain of Mig-6 directly binds to and inhibits the Rho family GTPase Cdc42. It has been shown that Mig-6 binds exclusively to the GTP-bound form of Cdc42 [40]. Interestingly, other proteins that contain a CRIB domain, such as Ack1 and PAK proteins, also interact with GTP-bound Cdc42 [28, 41]. Notably, the Ack1 CRIB domain has been shown to inhibit EGF-induced Cdc42 activation [28]. Since small GTPases such as Cdc42 can transit from an inactive GDP-bound to an active GTP-bound state, the interaction between Mig-6 and Cdc42 and other factors are likely to play an important role to inhibit Cdc42 activity. For example, Rho GDP dissociation inhibitors (RhoGDIs) have been shown to play a role in cell migration and cancer progression by preventing the exchange of a GDP nucleotide for GTP, thus inhibiting Cdc42 and other Rho family members, such as Rho and Rac [42–45].

Cdc42 is an important regulator of cytoskeletal organization that is best characterized for its role in regulating rearrangement of the actin cytoskeleton to promote filopodia formation [20]. Cdc42 exerts its biological effects primarily via group II p21-activated kinases (PAK), such as PAK-1 and PAK-4, which are majorly responsible for regulating actin cytoskeleton rearrangements [29, 30]. In this study, we show that Mig-6 inhibits EGF-mediated cell migration and filopodia formation. These results are consistent with the notion that Mig-6 functions majorly by directly inhibiting Cdc42, since abolishing this interaction completely abrogates the effect of Mig-6 in PAK-1 inactivation and in inhibiting EGF-induced cell migration. Notably, it has been shown that Mig-6 can inhibit Hepatocyte Growth Factor (HGF)-induced cell migration of hepatic cells [24]. Whether Cdc42 is involved must wait for further investigation.

Our analysis of clinical data indicates that Mig-6 expression decreases progressively in breast cancers of higher pathological stage, as well as in metastatic lesions, compared to primary tumors in prostate cancer. Consistent with these observations, we found that Mig-6 ablation enhances EGF-induced filopodia formation and cell migration. These results are in line with previous studies that found decreased Mig-6 expression in skin, ovarian, lung and pancreatic cancers [6, 19, 46]. Furthermore, reduced Mig-6 expression has been shown to correlate with poor prognosis in breast cancer patients [46]. It is plausible that Mig-6 down-regulation leads to up-regulation of Cdc42 activity. Interestingly, Cdc42 over-expression has been described in breast cancer, lung cancer and cutaneous melanoma, and its up-regulation correlates with testicular cancer progression [47–49]. These observations highlight the importance of this pathway in cancer metastasis, and open new avenues of research for investigating the relationship between increased growth factor signaling, Mig-6 expression and Cdc42 activity.

## MATERIALS AND METHODS

### Cell culture

Human non-small cell lung carcinoma H1299 and HEK-293T cells were maintained in Dulbecco's modified Eagle's medium (DMEM; Hyclone) supplemented with 10% fetal bovine serum (FBS, Hyclone) and 1% penicillin G/streptomycin sulfate. Human non-transformed mammary epithelial MCF-10A cells were maintained in 1:1 mixture of Dulbecco's modified Eagle's medium (DMEM) and Ham's F12 medium with reduced Ca^2+^ (0.04 mM; Invitrogen Inc.), 20 ng/mL epidermal growth factor (Invitrogen), 100 ng/mL cholera toxin (Sigma), 10 μg/mL insulin (Sigma), 500 ng/mL (95%) hydrocortisone (Sigma) and 5% of Chelex-treated horse serum (Invitrogen). Cells were maintained at 37°C in a humidified incubator under 5% CO_2_.

### Plasmid constructions, viral infections and RNA interference

Wild type and mutant Myc-tagged Mig-6 expression constructs were PCR amplified and sub-cloned separately into pLVX-IRES-zGreen1 lentiviral vectors using human Mig-6 as a template [15]. HA-and Flag-tagged WT Cdc42 was cloned into pLVX-PGK-Puro. Short hairpin RNA (shRNA) constructs against human Mig-6 have been described previously [15]. Lentivirus were amplified by transfecting HEK-293T cells with pMD2.G and psPAX2 packaging plasmids and the corresponding backbone plasmid using Lipofectamine 2000 (Invitrogen). Virus were collected 48 hours post-transfection, filtered, and used fresh for infection overnight in the presence of 10 μg/mL polybrene. Stable cells were selected as polyclonal populations by puromycin selection.

### Western blot analysis, immunofluorescence and immunoprecipitation

Cells were lysed in EBC_250_ lysis buffer (50 mM Tris-HCl, pH 8.0, 250 mM NaCl, 0.5% Nonidet P-40, 0.2 mM phenylmethylsulfonyl fluoride [PMSF], 2 μg/ml leupeptin, 2 μg/ml aprotinin, 50 mM NaF, and 0.5 mM Na_3_VO_4_). Proteins were resolved by SDS-PAGE, transferred to polyvinylidene difluoride membrane (Bio-Rad), and hybridized to an appropriate primary antibody and horseradish peroxidase-conjugated secondary antibody for subsequent detection by enhanced chemiluminescence (Millipore). Monoclonal antibodies specific for c-myc (9E10) and for Cdc42 (B8), and polyclonal antibody for actin (C-11) were purchased from Santa Cruz Biotechnology (Santa Cruz, CA, USA). Monoclonal antibody specific for Mig-6 (2440) were purchased from Cell Signaling (Danvers, MA, USA). Goat anti-mouse IgG-HRP (sc-2005) and goat anti-rabbit IgG-HRP (sc-2004) secondary antibodies for western blotting were obtained from Santa Cruz Biotechnology (Santa Cruz, CA, USA). For fluorescence microscopy, cells in chamber slides were fixed with 3.7% formaldehyde in phosphate buffer saline (PBS) and permeabilized with 0.1% Triton X-100 in PBS, prior to incubation with phalloidin (Sigma-Aldrich) in 1% bovine serum albumin (BSA) in PBS to visualize F-actin by fluorescence microscopy. Cells were counter-stained with DAPI (Beyotime) and fixed onto glass slides. Images were acquired using a Nikon Eclipse Ti-U fluorescence microscope equipped ELWD 0.52 camera (ELWD 40× / 0.60) and analyzed using SPOT Basic software. Filopodia were quantified by counting all cells and scoring cells with 20 or more filopodia in at least four random fields from at least three experiments. For examination of Cdc42-Mig-6 interaction, 293T cells were co-transfected with HA-Cdc42 and Myc-Mig-6. Cells were lysed in EBC_150_ buffer (same as EBC_250_ except for 150 mM NaCl) and precleared with mouse IgG-agarose prior to incubation with anti-HA antibody-conjugated agarose (Santa Cruz Biotechnology) for three hours at 4°C. Beads were then washed with PBS and eluted in 2× SDS sample buffer for five minutes at 95°C. Complexes were then separated by SDS-PAGE and immunoblotted using antibody specific for Myc or Cdc42, as indicated.

### Assays for cell migration

Cell migration was measured by *in vitro* wound-healing and by transwell assays. For wound-healing assay, stable cells expressing GFP as well as WT or mutant Mig-6 were grown to 90% confluency in complete growth media, then wounded with a plastic pipette tip. Cells were washed and photographed using a fluorescence microscope (Nikon Eclipse Ti-U). Cells were then incubated in DMEM containing 1% FBS in the presence or absence of 100 ng/mL EGF for 24 to 36 hours at 37°C in a humidified incubator under 5% CO_2_. Cells were then photographed again at the same location. Percent of wound closure was quantitated by measuring the wound at three sites at time zero, and then again after 24 hours. Mean closure values from three independent experiments performed in duplicate were used to calculate reported results. Transwell assays were performed using 6.5 mm, 8.0 μm-pore polycarbonate membrane transwell inserts (BD Biosciences, San Jose, CA, USA). Briefly, 0.5–1.0 × 10^5^ cells were suspended in serum-free DMEM media and seeded into the inner chamber. The outer chamber contained complete growth media supplemented with 100 ng/mL EGF. Cells were incubated for 8 or 24 hours and then non-migrating cells on the inside of the membrane were carefully removed with a cotton swab, while migrating cells on the outside side of the membrane were fixed and stained with 0.5% crystal violet in 70% ethanol. For each transwell assay, we counted numbers of cells under an optical microscope from randomly chosen 5 fields of each sample in duplicate. Three independent experiments were performed followed by statistical analyses and presented as means and SE.

### Active Cdc42 detection

Active Cdc42 level was detected by using Active Cdc42 Detection Kit (Cell Signaling Technologies, #8819). Briefly, GST-PAK1-PBD fusion proteins were used to bind GTP-bound Cdc42, which was then immunoprecipitated with glutathione resin and detected by western blotting.

### Bioinformatic analysis of gene expression

Oncomine^TM^ (Compendia Bioscience, Ann Arbor, MI) was used for analysis and visualization.

## SUPPLEMENTARY MATERIALS FIGURES


