# Supplementary material for: Inhibition of Cdc42 is essential for Mig-6 suppression of cell migration induced by EGF
Source: Oncotarget. 2016 Jun 21;7(31):49180–93. doi: 10.18632/oncotarget.10205 (PMC5226500; doi:10.18632/oncotarget.10205)
Supplement: Supplementary file 1 [file oncotarget-07-49180-s001.pdf]

Inhibition of Cdc42 is essential for Mig-6 suppression of cell migration induced by EGF

Supplementary Materials

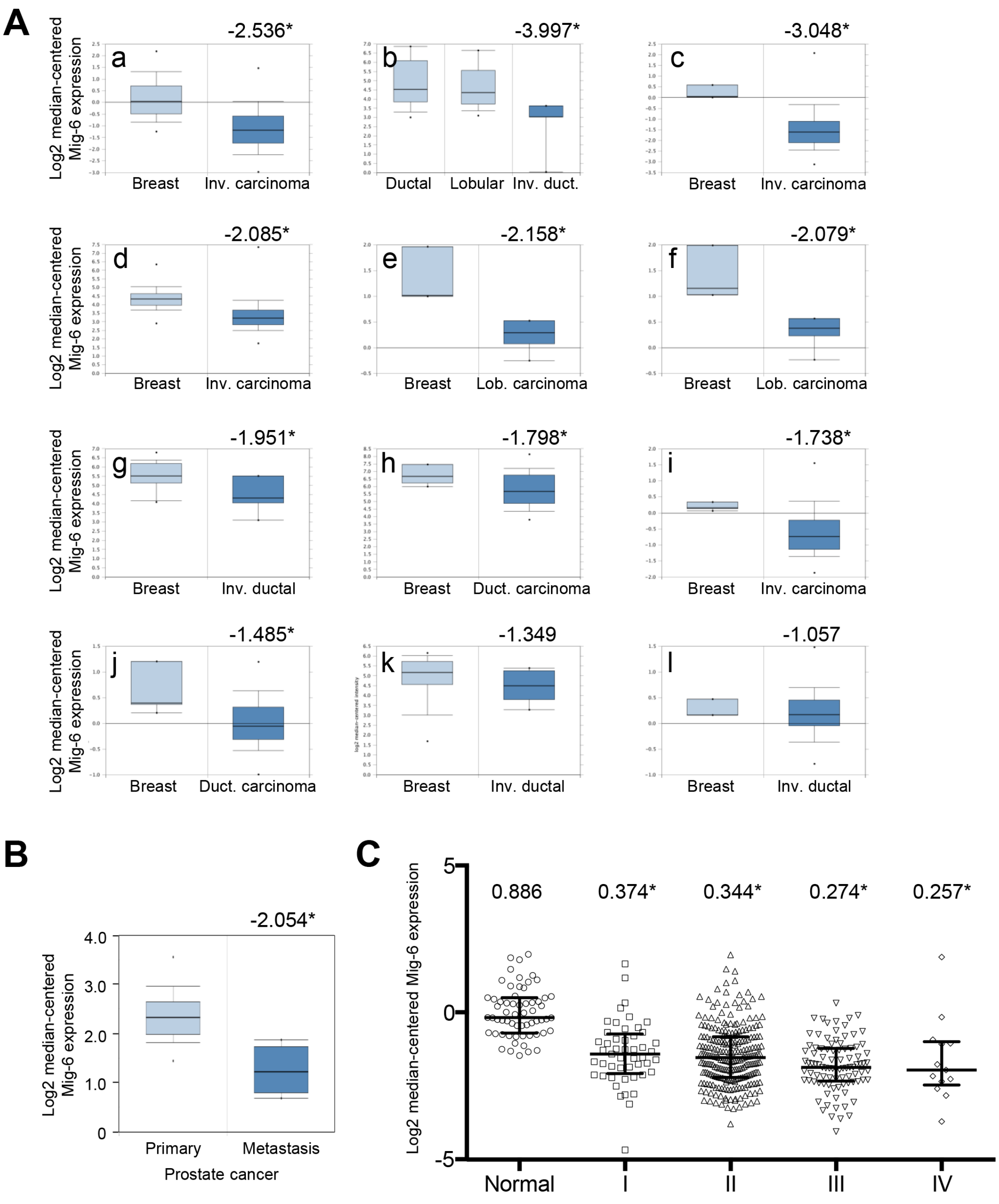

Supplementary Figure 1: Mig-6 expression is decreased in advanced human cancers. (**A–B**) Box plots representing Mig-6 gene expression from microarray analysis of breast cancer biopsy samples (A) and primary versus metastatic prostate cancer (B). The top and bottom of each box represent the first and third quartile, and the band inside the box represents the median value. Error bars represent one standard deviation above and below the mean value. Fold changes compared to control are indicated above each box plot. **P* < 0.05. a: TCGA Breast; b: Turashvili Breast; c: Finak Breast; d: Curtis Breast; e: Perou Breast; f: Sorlie Breast; g: Karnoub Breast; h: Richarson Breast; i: Gluck Breast; j: Sorlie Breast 2; k: Ma Breast 4; l: Zhao Breast. (**C**) Mig-6 expression in breast cancer tumor biopsies classified pathologically by stage. Median expression for each group is indicated. **P* < 0.05.

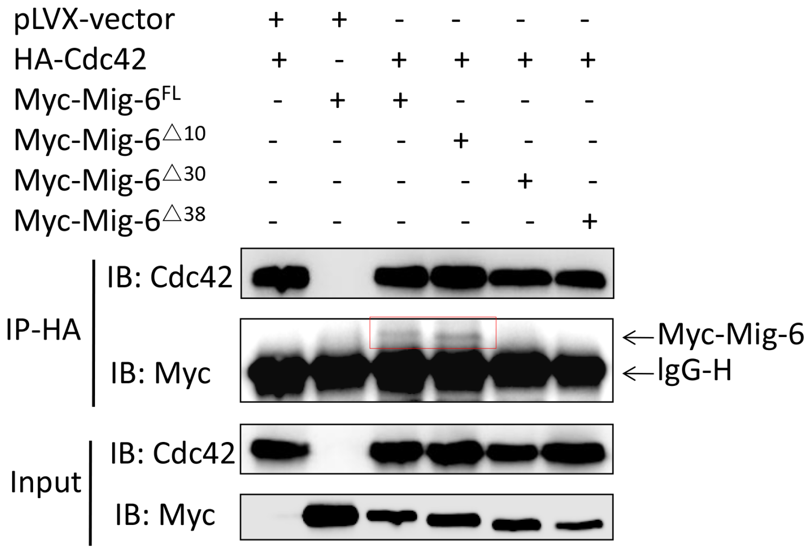

Supplementary Figure S2: Residues 11-30 in the CRIB domain of Mig-6 are essential for Mig-6 binding to Cdc42. Cell lysates from 293T cells co-transfected with HA-Cdc42 and Myc-Mig-6 (FL) or a deletion mutant were subjected to IP-western analysis, as shown. Note that Δ30 and Δ38 were unable to interact with Cdc42.

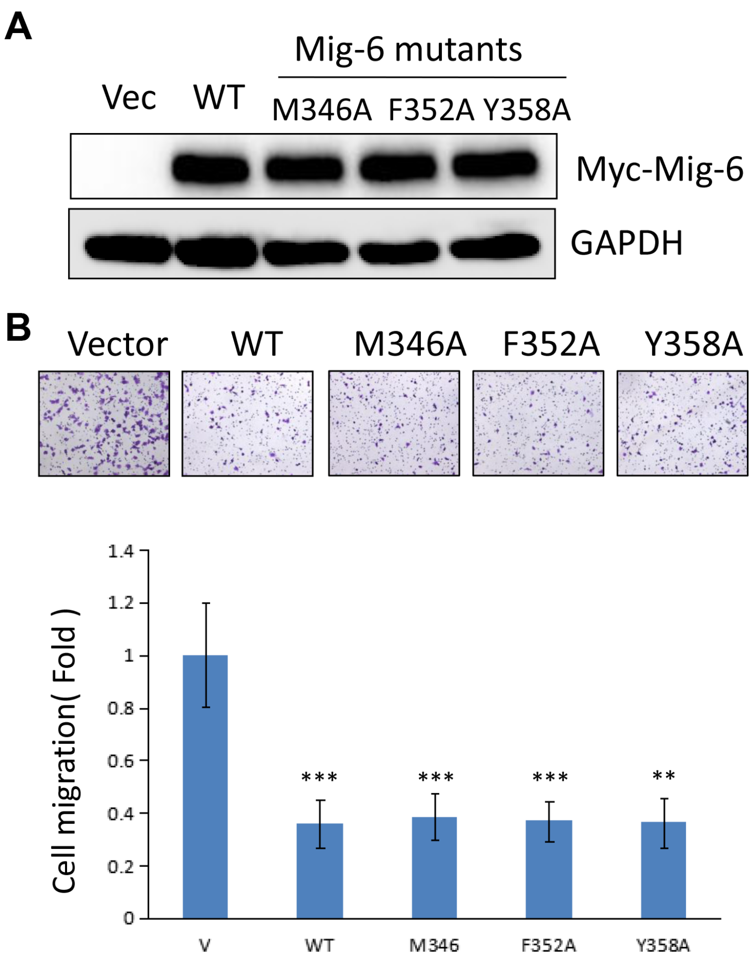

Supplementary Figure S3: Mig-6 inhibit cell migration independently of EGFR binding. (**A**) H1299 cells were stably infected with lentivirus expressing wild type (WT) or EGFR-binding deficient mutant Myc-tagged Mig-6(M346A, F352A, Y358A). Whole cell lysates were subjected to western blotting as shown. (**B**) Stable H1299 were subjected to transwell assays in the presence of EGF for 8 hours. Results from three independent migration assays were quantitated and presented as means and SD. ****P* < 0.001, ***P* < 0.01.

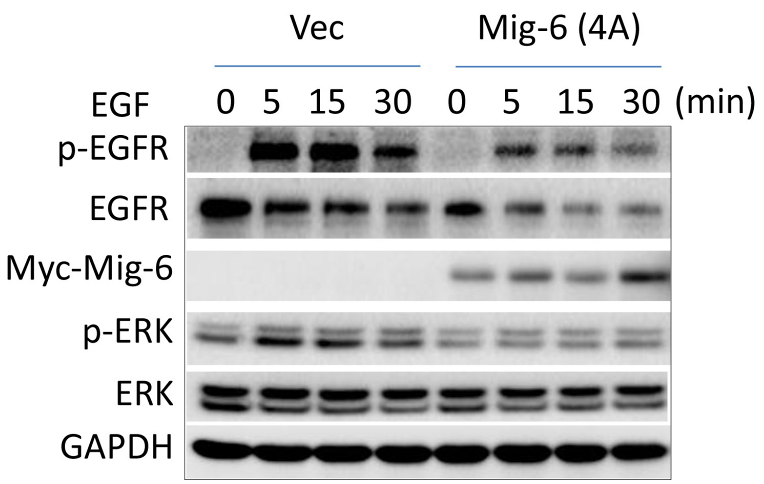

Supplementary Figure S4: Mig-6(4A) inhibited EGFR phoshporylation and promoted its internalization. H1299 cells stabling expressing Myc-tagged Mig-6 4A mutant (Mig-6(4A)) or GFP alone (VEC) were treated with 100ng/ml EGF for indicated time after 12 hours of serum starvation. Cell lysates were subjected to western as shown.

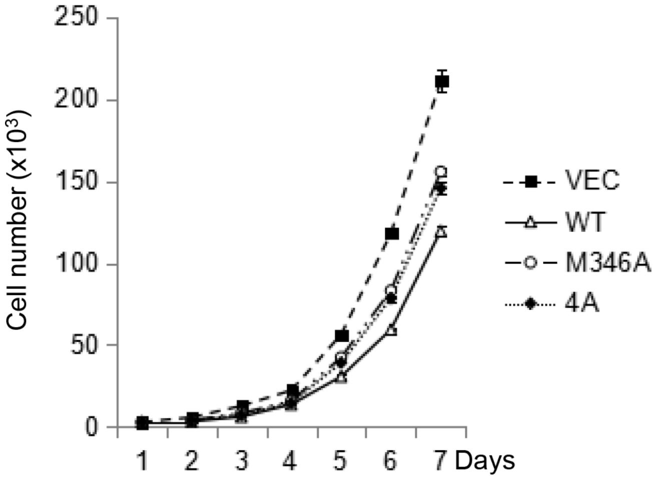

Supplementary Figure S5: H1299 stable cells were grown in DMEM containing 10% FBS in the presence of EGF (100 ng/mL). Cell numbers were counted every 24 hour for 7 days. Two independent experiments in duplicate were performed.

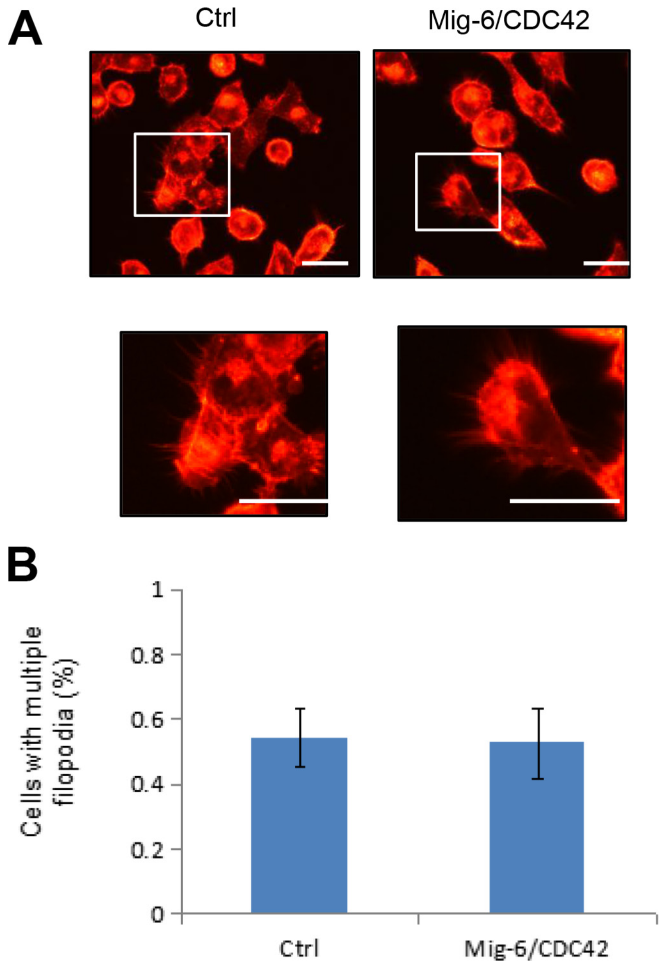

Supplementary Figure S6: Stable cells exhibiting filopodia were visualized by fluorescent microscopy. F-actin was visualized with phalloidin (red), and nuclei was counter-stained with DAPI (blue). Representative images (and insets) from three independent experiments performed in duplicate are shown. Scale bars = 50 µm. (E) Percentage of cells with multiple filopodia (defined as cells exhibiting at least 20 filopodia) were quantified and presented as means and SE from three independent experiments. *$P < 0.05$.
